# Continued Success of the Kousso in Promoting the Expulsion of the Tape-worm

**Published:** 1850-08

**Authors:** 


### PATHOLOGY AND PRACTICE OF MEDICINE.

### KING'S COLLEGE HOSPITAL.

*Continued Success of the Kousso in promoting the Expulsion of the Tape-worm.*—In former numbers of the Lancet, (March 16, 1850, and April 20, 1850,) cases were noticed in which the Kousso was found very efficacious for procuring the expulsion of the tænia solium. This plant is now acknowledged to be so useful in tape-worm, that it seems almost unnecessary to adduce new cases; we shall, however, just sketch a few of those which were lately benefited by the Kousso, as they present various features of interest.

The first case, as taken from Mr. Jordan's notes, runs as follows:—Rebecca R., aged 22, is a native of Wapping; she went to Devonport when seven years of age, but only stayed there about a fortnight; with this exception she has constantly lived in town, generally at Wapping, but about eighteen months ago she spent a year at Peckham. Patient's sister, who has been dead nine years, also suffered from tape-worm, which remained upon her to the time of her death. Patient likewise knows of a neighbor of hers in Wapping, close to her own home, who suffers from the tænia. This latter person and the above-mentioned sister are the only people she knows to be thus affected. The water is supplied by the New River Company to the whole neighborhood.

Patient was quite healthy until about two years ago, since which time she has had great pain in the side and stomach; her appetite was good, but she used to feel sick on first getting up; she had, however, no idea that she harbored a tape-worm until a week before Christmas, when she first passed joints of it, and from that period, such joints have been passed almost every day.

Twice since she first noticed the joints she has passed long pieces of the worm, once after opening medicine, the other time without any such agency. She has never taken any turpentine nor any other remedy expressly for the worm.

Patient was admitted under the care of Dr. Budd, and took the Kousso at half-past nine in the morning, the day after her admission; and, after taking a dose of castor oil in the middle of the day, the worm was passed with a motion at a quarter to five in the afternoon. This entozoon was nearly three yards in length, and the narrow segments approaching to the head were attached to it, though not the head itself. The medicine gave patient a slight feeling of sickness, which soon went off again. Her appetite was bad on the day she took the Kousso, and she felt weak. With the exception of the tape-worm patient seems to have generally had good health; she has only a slight cough. Her mother and sister died of phthisis, but patient's appearance is remarkably florid and healthy. The day after admission, this woman left the hospital in good condition, without passing any more of the worm.

The second case was admitted under the care of Dr. Todd. The subject is a young woman, native of Scotland, *four months advanced in*

*pregnancy.* She complained to Mr. Steele, the house-physician, that she was in the habit of passing long *round* worms, but when she brought the joint, which she had lately evacuated, they were found to be pieces of the tænia solium. When the nature of the worm was ascertained, the patient was admitted into the house, and took the Kousso in the morning; at seven in the evening she went home, and a quarter of an hour after she had reached her residence, she passed five yards of the worm.

The third case was sent to Dr. Todd from the country. The patient is a middle-aged woman, residing at Bow, who took the Kousso at three o'clock in the afternoon, and left the house immediately afterwards, promising to bring the worm as soon as she should evacuate it. The next morning she brought a tape-worm measuring about six yards in length.

The fourth case, who was admitted under the care of Dr. Budd, is that of a man, about 46. His health has, in general, been pretty good; last winter, however, he was attacked by cholera, and treated in King's College Hospital. Whilst laboring under this disease, patient did not pass any joints of the tape-worm, though previous to his being visited by the epidemic he had now and then evacuated portions of the tænia. When convalescent, he took some oil of turpentine, and by the agency of this medicine he voided a few joints. From that period he continued passing joints, and was admitted under the care of Dr. Budd, May 3, 1850. Patient took the Kousso in the morning, and had two doses of house medicine in the course of the day. At six o'clock in the evening, he passed a tape-worm of a very great length, since it measured nearly ten yards. The next day he voided a piece, six inches long, which came evidently from very near the head. It is to be regretted, as we stated before, that this medicine is so expensive; still, when it is considered how rapidly and effectually it promotes the evacuation of the tænia, the 17*s.* 6*d.* can hardly be looked upon as a high price; the more so, as in hospital practice, the patients need stay in the house but a short time. It will be extremely interesting to keep an eye upon these patients, in order to ascertain whether the benefit is of a lasting or temporary kind.—*Lancet.*

